# An Artificial Intelligence-Based Tool for Data Analysis and Prognosis in Cancer Patients: Results from the Clarify Study

**DOI:** 10.3390/cancers14164041

**Published:** 2022-08-22

**Authors:** María Torrente, Pedro A. Sousa, Roberto Hernández, Mariola Blanco, Virginia Calvo, Ana Collazo, Gracinda R. Guerreiro, Beatriz Núñez, Joao Pimentao, Juan Cristóbal Sánchez, Manuel Campos, Luca Costabello, Vit Novacek, Ernestina Menasalvas, María Esther Vidal, Mariano Provencio

**Affiliations:** 1 Department of Medical Oncology, Puerta de Hierro-Majadahonda University Hospital, 28222 Madrid, Spain; 2 Faculty of Health Sciences, Francisco de Vitoria University, 28223 Madrid, Spain; 3 Department of Electrical Engineering, NOVA School of Science and Technology, Universidade Nova de Lisboa, 2825-149 Lisbon, Portugal; 4 Department of Mathematics and CMA, NOVA School of Science and Technology, Universidade Nova de Lisboa, 2825-149 Lisbon, Portugal; 5 Chronobiology Lab, Department of Physiology, College of Biology, Mare Nostrum Campus, University of Murcia, 30100 Murcia, Spain; 6 Biomedical Research Institute of Murcia (IMIB)-Arrixaca, 30120 Murcia, Spain; 7 Accenture Labs, D02 P820 Dublin, Ireland; 8 Data Science Institute, NUI Galway, H91 A06C Galway, Ireland; 9 Centro Tecnología Biomédica, Universidad Politécnica de Madrid, 28223 Madrid, Spain; 10 TIB Leibniz—Information Centre for Science and Technology, 30167 Hannover, Germany

**Keywords:** artificial intelligence, data integration, cancer patients, patient stratification, precision oncology, decision support system

## Abstract

**Simple Summary:**

Cancer is associated with significant morbimortality worldwide. Although significant advances have been made in the last few decades in terms of early detection and treatment, providing personalized care remains a challenge. Artificial intelligence (AI) has emerged as a means of improving cancer care with the use of computer science. Identification of risk factors for poor prognosis and patient profiling with AI techniques and tools is feasible and has potential application in clinical settings, including surveillance management. The goal of this study is to present an AI-based solution tool for cancer patients data analysis and improve their management by identifying clinical factors associated with relapse and survival, developing a prognostic model that identifies features associated with poor prognosis, and stratifying patients by risk.

**Abstract:**

Background: Artificial intelligence (AI) has contributed substantially in recent years to the resolution of different biomedical problems, including cancer. However, AI tools with significant and widespread impact in oncology remain scarce. The goal of this study is to present an AI-based solution tool for cancer patients data analysis that assists clinicians in identifying the clinical factors associated with poor prognosis, relapse and survival, and to develop a prognostic model that stratifies patients by risk. Materials and Methods: We used clinical data from 5275 patients diagnosed with non-small cell lung cancer, breast cancer, and non-Hodgkin lymphoma at Hospital Universitario Puerta de Hierro-Majadahonda. Accessible clinical parameters measured with a wearable device and quality of life questionnaires data were also collected. Results: Using an AI-tool, data from 5275 cancer patients were analyzed, integrating clinical data, questionnaires data, and data collected from wearable devices. Descriptive analyses were performed in order to explore the patients’ characteristics, survival probabilities were calculated, and a prognostic model identified low and high-risk profile patients. Conclusion: Overall, the reconstruction of the population’s risk profile for the cancer-specific predictive model was achieved and proved useful in clinical practice using artificial intelligence. It has potential application in clinical settings to improve risk stratification, early detection, and surveillance management of cancer patients.

## 1. Introduction

Artificial intelligence (AI) has contributed substantially in recent years to the resolution of different biomedical problems including cancer. Cancer is the leading cause of death in developed countries, and it is estimated that the number of cases will increase further in aging populations [1]. However, advances in early detection and improvements in therapeutics (not only in early disease but also in metastatic settings) contribute to decrease cancer mortality and increase the amount of cancer survivors [2]. Evidence-based medicine, based on a patient-centric approach, is rapidly replacing experience-based medicine. In this sense, an increasing number of studies indicate that AI could revolutionize medicine, being the key driver of the transformation of healthcare to precision and personalized medicine. Specifically, in oncology, which is rapidly becoming data-intensive, we are only starting to understand the practical implications of AI [3]. Thus, there are major barriers for AI implementation in oncology, such as biased and heterogeneous data, a lack of standardized collection and research reporting, insufficient clinical validation, or outdated regulatory and legal frameworks [3,4,5,6].

Big data is extremely useful in the actual digitization of the healthcare stage. Traditional software approaches are not suitable to cope with the challenges imposed by the digitalization of healthcare which is dramatically changing clinical workflow. Both healthcare providers and clinicians are now drowned with extremely large and fast generated, heterogeneous, structured, and unstructured data sets with questionable value and veracity which are too complex to be dealt with by traditional software [7]. Automated algorithms can help to process this kind of data and extract meaningful patterns that provide practical knowledge, changing the way in which treatments outcomes are evaluated, patients are classified, and diseases are studied [8].

In the past few years, the terms AI and ‘machine learning’ (ML) have involved the achievement of several important medical advances with their application [7]. What we, as clinicians, ask ourselves is the following: how can their application affect the practice of medicine? In oncology, the obvious question is ‘How will AI improve the outcomes of patients with cancer?’.

In the last few decades, major advances in technology have produced large-scale, multidimensional data for cancer research [8,9]. Previously, cancers were simply characterized by anatomical site, pathology, and histological type. Nowadays, we acknowledge cancer as a multifactorial disease, wherein each type of cancer is biologically distinct requiring its own unique treatment and management. New cancer diagnoses are currently focused in more complex and precise methods such as measurement of molecular features (DNA, RNA, or proteins) to accurately characterize the tumor and match the individual to a molecularly informed and targeted treatment plan [10]. Even after treatment is complete, the combination of clinical, pathology, molecular, treatment, and response data can be used to generate evidence necessary to help numerous patients [2,7].

Analysis and sharing of these kinds of clinical data has become increasingly paramount as our knowledge of the extreme heterogeneity of cancer continues to grow. The application of AI algorithms has the potential to transform health and healthcare delivery by helping solve complex problems, such as quality of life improvement, increase in the chances of survival, and maximizing safety [11,12,13]. Common applications of AI algorithms in health care include identifying conditions, events, risk factors, associations, and patterns of difference or similarity, which can be used to support clinical decision making, improve treatment outcome, evaluate cost-effectiveness of tests, increase efficiency, and facilitate discovery [14].

This exceptional complexity inherent to oncological diseases and oncological patients presents an opportunity for AI to impact oncology-related problems. However, few AI tools have yet had a significant and widespread impact in oncology [15]. The goal of this study is to present an AI-based solution tool for oncology problems validated at a medical and research institution in Spain, proposing steps based on our results and experience that will help foster the development and deployment of AI tools in routine clinical practice based on evidence.

## 2. Materials and Methods

### 2.1. Patients and Data

This is a hospital-based retrospective registry that updates prospective follow-up data of a total of 5275 patients diagnosed since 2008 at Medical Oncology Department at Hospital Universitario Puerta de Hierro-Majadahonda (HUPHM) with non-small cell lung cancer (NSCLC), breast cancer or non-Hodgkin’s lymphoma, regardless of their treatment, sex, or age. The last follow-up or vital status information was updated in December 2021. The study was approved by the Ethics Committee at HUPHM (No. PI 148/15) and was carried out in accordance with the Helsinki Declaration.

This registry represents a large representative body of cancer outcomes, allowing for generalizability of findings, making it an excellent source for reconstructing cancer-specific risk profiles of the population. These data are integrated in the platform along with wearables data, quality of life (QoL) questionnaires data, and genomic data.

### 2.2. Decision Support Platform (DSP)

**Figure 1:**
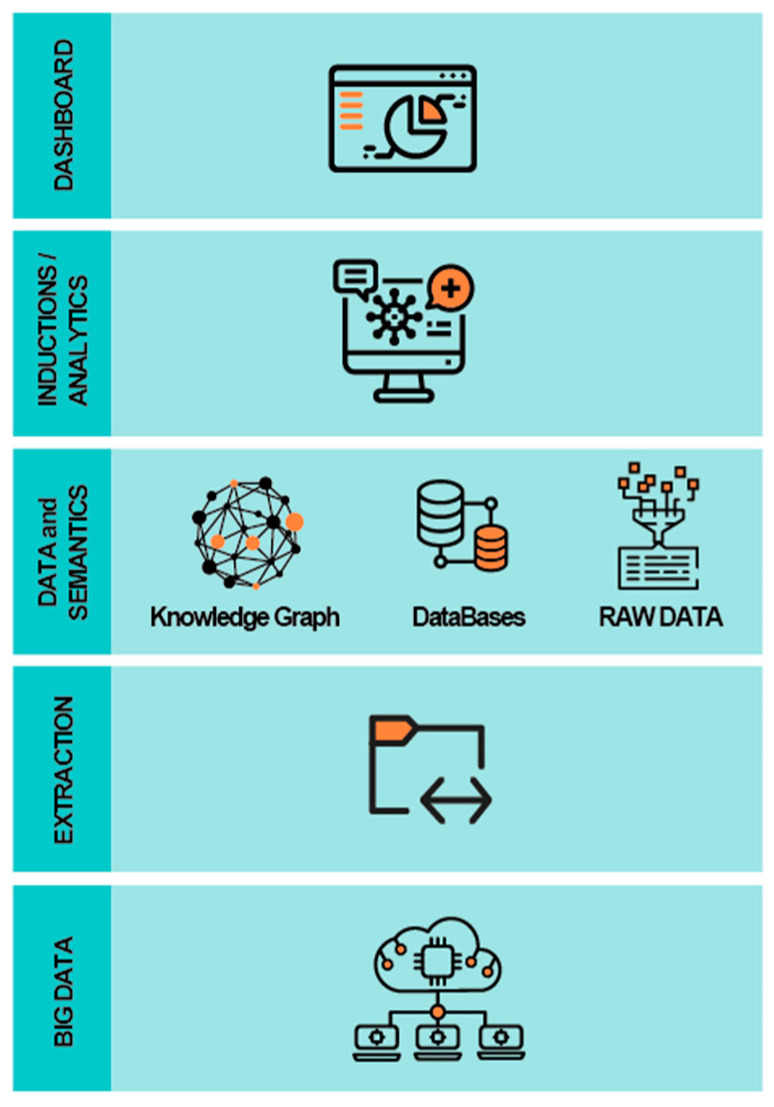
The CLARIFY platform system architecture.

CLARIFY (Cancer Long Survivor Artificial Intelligence Follow-Up), a European project supported by the EU Horizon 2020 Research and Innovation Program (grant agreement No. 875160), has developed a System Architecture that allows centralizing anonymized clinical data and providing an efficient methodology to share it among clinicians and researchers (https://www.clarify2020.eu/ accessed on 1 August 2022). CLARIFY Decision Support Platform (DSP) consists of a responsive Web application, capable of extracting and displaying information, from several sources of data, such as spreadsheets, APIs, databases, files, reports, wearable monitoring devices and others, in a coherent, structured and readable way, as well as organize it and present it according to the end-user needs (Figure 1). The DSP provides quick and insightful answers to the end-users’ questions in an easy-to understand visual format.

Cancer patients’ clinical data are collected in real-time from a variety of sources and have the greatest value for efficient modeling of cancer risk and recurrence. It shows to the clinical user either information about individual patients or about the whole population and produces descriptive statistics that enable description and analysis from the data sets. With this tool, it is possible to describe the characteristics of sample data and include some common statistical tools and procedures such as mean, variance, and segmentation based on specific variables, along with survival analysis with Kaplan–Meier estimates and Cox regression model and, for event estimation, logistic regression. Integrated data include clinical data from the electronic health records (EHR) from 5275 patients (2250 NSCLC, 2025 Breast Cancer, and 1000 Lymphomas), that include more than 11,000 health clinical processes, more than 700,000 values, 350,000 clinical notes, more than 900,000 clinical reports, and 1,500,000 tests requests, among other figures; data from 350 patients monitored with the wearable device that produced around 1,000,000 variable values per patient, and data from QoL questionnaires from 650 patients.

The DSP does not store data, entailing that all data are retrieved in real-time over the data stored in the project infrastructure, as per end-user requests and according to the user’s profile. All information that is presented is extracted using queries, web services, or API requests.

### 2.3. Technical Description of the CLARIFY Platform

**Figure 2:**
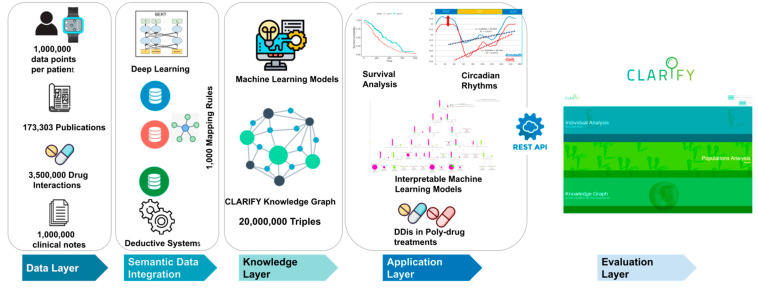
Data integration layers in CLARIFY DSP.

In order to create a robust, scalable, and fully flexible system with low response time and high availability for its users, the architectural general principles considered in the development of the CLARIFY architecture were availability, fault tolerance, maintainability, performance, robustness, scalability, security, and usability. Moreover, the architecture is based on the structure of Big Data Europe (BDE), an easy-to-deploy, easy-to-use and adaptable (cluster-based and standalone) platform for the execution of big data components and tools such as the ones required for the CLARIFY platform. As a result, the CLARIFY platform allows performing a variety of Big Data flow tasks such as message passing, storage, analysis and publishing, and its architecture implements a layered architecture pattern consistent with modern distributed systems architecture principles (Figure 2).

The user experience was a key factor in the development of the user interfaces. User high-level requirements were focused in the development of different layers: a Big Data layer that integrates heterogeneous sources of data (whose volume, variety, veracity, some velocity and clearly value classify it as Big Data); an extraction layer, responsible for the extraction of different kinds of data (anonymized personal data from electronic health records, wearable devices, questionnaire answers and genomic datasets); a data and semantics layer for storage of large and complex data in various stages of processing; an induction/analytics layer, devoted to the analysis of the different data sources based on the requirements presented by the end-users (the clinicians), where models were built to be applied to specific patient cases or communities with similar characteristics; and the dashboard, the final layer which aims to support the decisions of the clinicians.

### 2.4. DSP Functionalities

#### 2.4.1. Descriptive Statistics

**Figure 3:**
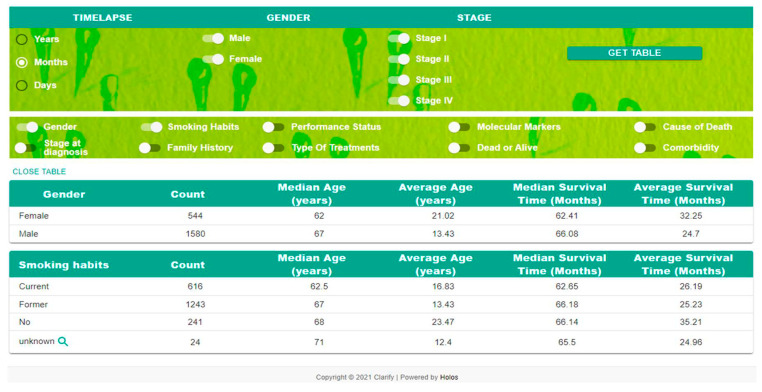
Descriptive statistics of the lung cancer patient’s dataset.

The DSP produces some basic descriptive statistics that enable description and analysis from data sets. With this tool, it is possible to describe the characteristics of sample data (Figure 3). Some common statistical tools and procedures include the following: mean, median, variance, and segmentation based on specific variables.

#### 2.4.2. Survival Analysis

A Kaplan–Meier estimator was used to fit the estimates regarding one covariate, which allows plotting the survival curves for each value of that covariate (e.g., for the covariate Stage of Diagnosis, one may plot, for each category, the correspondent survival curve). The Cox Regression Model is a multivariate model that allows for the identification of the characteristics of the patients that most influence the survival time. For this, after defining a baseline patient (baseline characteristics) the remaining values for each covariate are compared and the hazard ratio is estimated. This model allows one to identify which covariates have a positive or negative impact on survival. The model also allows one to understand which variables are the most significant when compared with the baseline patient profile.

## 3. Results

### 3.1. Application of the CLARIFY DSP

This computational infrastructure is able to extract knowledge from data sources of different nature (e.g., clinical notes, patient opinions, genomic data, scientific publications, and wearables, allowing clinicians to analyze multiple factors that will help stratifying patients by risk, in order to implement a personalized follow up care program aiming to make a significant impact in the patients’ wellbeing and QoL). It is worth noting that the focus should not be on a single risk, as the patient must be stratified according to the individual risk of developing any of the problems that affect the long survivors. This is either carried out through clinical monitoring or by means of tests that imply a high cost in these cancer survivors.

The data integration achieved by the CLARIFY platform provides clinicians with a novel tool for real time data access and analysis, allowing cancer patient profiling, cohort descriptive analysis based on selected parameters, or survival studies based on specific pathological and treatment features. It also allows clinicians to compare the behavior and outcomes of their patients with larger series or clinical trials and may assist clinicians in their daily clinical practice by assessing risk of relapse in a certain patient profile, perform a stratification of a selected patient or group of patients, and determine their behavior in terms of certain aspects of the disease that cannot be analyzed in any other way, such as response or not to a certain treatment or utility of follow-up tests. As a proof of concept, we aim at developing real-time EHR-based algorithms to estimate oncology patients’ risk of short-term mortality prior to chemotherapy initiation based on structured and unstructured EHR data, which are theoretically applicable to any EHR. In this sense, this DSP is being tested in other hospitals in order to validate the tool and the results obtained at HUPHM, allowing for comparison to made of different cancer patient cohorts and behavior of different groups within the same pathology or between different pathologies, which could be extremely useful to oncologists at the point of care.

This platform allows clinicians to obtain immediate and personalized information of a selected patient or a whole cohort and to elaborate models based on statistical relational learning and explainable AI techniques to predict patient-specific risk of developing complications and toxicities secondary to their cancer treatments based on their clinical profile. These models will help clinicians to make evidence-based treatment and post-treatment decisions in a way that it is not possible with any existing approach. By using these AI techniques, we will be able to exploit large amounts of clinical information integrated in an interactive user that will facilitate early discovery of risk factors that may deteriorate a cancer patient’s condition during and after treatment. It also allows us to examine the effect of multidisciplinary interventions in order to personalize their follow-up by better assessment of their needs and eventually improve their quality of life, wellbeing, and outcome.

In this way, the DSP represents a novel decision support system that meets clinicians’ needs and can uncover hidden properties and associations that enhance the knowledge of clinicians and public health policy makers. Beyond the effectiveness of the CLARIFY DSP services, stakeholders can also rely on this web service-based infrastructure and deploy applications tailored to specific-user needs and augment further the possibilities of defining public health policies, taking into account the scalability of the application (i.e., that the infrastructure is ready to scale and handle the data as eventually reaching a Big Data paradigm).

### 3.2. Artificial Intelligence for Predicting Clinically Relevant Parameters in Survival

Exploring the vast data captured by EHR has allowed us to identify patterns of clinically relevant parameters using individual and historical data from clinical notes [15]. EHRs organized data in a standard structure which can be processed using AI-based natural language processing algorithms. These can be cost-effective and straightforward tools used to support medical decision making. AI-based algorithms that predict survival have been developed for many cancer types, including breast, prostate, and lung cancers [15,16], and have shown better accuracy than conventional analytic approaches [16]. This may be due to improving fit for variables with nonlinear relationships, and thus becoming more applicable to real-life data and settings. We developed a prognostic model for early stage lung cancer patients within the DSP CLARIFY platform where EHR information is retrieved through multiple layers of neural networks allowing clinically-relevant analyses. The prognostic model identified a high-risk profile defined as follows: males over 70 years old, former smokers, stage II, ECOG-PS 2, treated with surgery. The identified features for the low-risk profile were being a woman between 20 and 50 years old, being a non-smoker, having been treated with surgery and adjuvant chemotherapy, and having an ECOG-PS 0 [17].

Risk-stratification, treatment complications and outcomes, or survival are some of the prognostic parameters that can be accessed using AI algorithms. Thus, the applicability of such models in real-life settings requires overcoming great data-related obstacles such as standardization, technological infrastructure, and organizational data culture.

### 3.3. Artificial Intelligence for Predicting Disease Recurrence

The risk of disease recurrence after curative treatment can been predicted using AI models. In certain types of cancer, the use of AI for recurrence prediction has shown increased accuracy, allowing for further support clinical follow-up optimization plans [5,7]. We developed a machine-aided lung cancer tumor recurrence prediction tool integrated in the CLARIFY DSP based on graph machine learning and explainable artificial intelligence methods to add an extra context clinicians can use when deciding how much to trust the prediction score. This machine-learning model predicts risk of recurrence based in the use of example-based methods that highlight previous patients with similar profiles that influence the prediction. We modeled the lung cancer clinical data as a knowledge graph [18]. Such information covers the selected 1348 early-stage NSCLC patients and their clinical data, and does not include any additional datasets (e.g., drugs, publications, genes). We predict the probability of recurrence of a patient and report AI-generated explanations for each approach. These graph machine-learning models may prove to be an appropriate auxiliary tool in the TNM classification systems that would optimize patient’s prognosis in lung cancer as they may enable objective and reproducible prediction of recurrence and disease outcome in patients with early-stage NSCLC [18].

## 4. Discussion

AI is becoming a useful and necessary tool in oncology to integrate all of the different features related and non-related to the disease that impact the patient’s outcome, quality of life, and survival. It can also provide accurate estimates of a patient’s risk for complications, including rehospitalization, cancer recurrence, treatment response, treatment toxicity, and mortality [17,19,20,21,22]. Enhanced predictions may offer potential advantages, including facilitating treatment planning, guiding population management efforts, and fostering discussions about goals of care [14,23]. Predictive analytics may aid the delivery of oncology treatments to populations that are disadvantaged or underrepresented in clinical trials for whom it is challenging to apply evidence-based medicine [24]. For example, among older adults, AI predictive analytics may help oncologists anticipate problems that have not been reported previously or identify risk factors for chemotherapy toxicity that are overlooked in daily practice given that these patients are usually not eligible for clinical trials.

Risk integration models and molecular tools to design an individual treatment planning with psychosocial support, counselling on genetic predisposition, fertility, sexual health, and socio-economic impact (among others) need to be developed in order to improve the survival and quality of life of cancer patients, which is currently lacking [25].

In this line of thinking, precision medicine is nowadays a growing field [26]. Many of the technologies that will be needed to meet the goals of precision medicine are in the early stages of development or have not yet been developed. Also, efficient ways to store large amounts of patients’ data in databases remains an open issue. In addition, ways of analyzing these data efficiently and combining them with other available datasets need to be studied [27]. While the integrated analysis of large datasets becomes more necessary and common, new data models are needed to collect, share, integrate, and interpret good quality and multi-dimensional data while protecting the patient’s privacy. Further, managing the data complexity generated by the dominant characteristics of biomedical big data (i.e., volume, velocity, and variety) is a major challenge. Currently, health and biomedical data are stored within institutions in innumerable, incompatible, and unconnected databases, so data quality is uneven. Data quality, integration and interpretation is key for data-driven approaches [28]. The more data integrated, the better the results once AI algorithms are applied [17].

A key challenge in medicine is the precise classification of patients and the development of tailored therapy plans which are optimal for each patient and expected to improve their outcome. Precision medicine can transform the delivery of healthcare to patients by developing a personalized risk-stratified follow-up care [5,17]. In this way, healthcare systems will evolve from a reactive “one-size-fits-all” towards systems that deliver predictive, preventive, and personalized care. These personalized approaches can only come from evidence-based medicine and are particularly promising in areas such as oncology. One of the most promising expectancies for AI is the possibility to integrate different and composite data derived from multi-omics approaches to oncologic patients. The promising tools of AI could be the only able to manage the big amount of data from different types of analysis, including information derived from DNA and RNA sequencing. Also, in addition to the terabytes of data being produced by EHRs, the use of PREMs and PROMs, or the incorporation of telemedicine with wearable devices in the clinical practice, AI tools that allow clinicians to analyze real-world data from their patients are needed [11,12]. In this sense, patterns and profiles may be obtained from specific patients or populations and AI-based predictions and algorithms can assist clinicians in medical decisions based on real-world medical evidence. In this sense, CLARIFY DSP already integrates the information on biomarkers that determine targeted therapy, quality of life questionnaires results, and wearable monitoring data, which helps clinicians to obtain a bigger picture of the global health status of the patient and make decisions based not only in clinical trials results but also on the real-life settings of each patient. A subsequent improvement in the platform will be to include results from genomic analyses, sequencing data, and less common mutations.

Ultimately, this AI-based approach is expected to lead to better health outcomes, improved prediction of morbidity and mortality risk, personalized risk-stratification follow-up care on patient function, health care utilization, costs and clinician satisfaction, improve supported self-management, and reduction in toxicity by earlier detection and mitigation of adverse effects, with the aim of better meeting the needs of survivors [29].

Clinicians will need to explain the role that AI has in their reasoning and recommendations [13] and, at the same time, empower patients and caregivers, promoting behavioral changes, facilitating self-monitoring of symptoms, improving their educational level, and recognize patients as co-creators of their own health with AI-based solutions.

## 5. Conclusions

This study reveals a feasible data-driven artificial intelligence solution for cancer-specific survival estimation, risk stratification, and potentially the follow-up management of cancer patients, which is applicable to different pathologies (lung, breast, and non-Hodgkin’s lymphoma), using data from multiple institutions throughout Spain.

This data integration provides clinicians with a novel tool for real time data access and analysis, allowing patient profiling, cohort descriptive analysis based on selected parameters, or survival studies based on specific pathological and treatment features. These analyses may support clinicians to incorporate improvements in the daily clinical practice, since the platform can provide real-time feedback to health professionals and patients by assessing risk of relapse in a certain patient profile, perform a stratification of a selected patient or group of patients, and predict their behavior in terms of certain aspects of the disease that cannot be analyzed in any other way, such as response or not to a certain treatment or utility of follow-up tests.

## Data Availability

The data presented in this study are available in this article.
